# Autoencoding-Assisted Quantum Cloning Machine

**DOI:** 10.3390/e28050563

**Published:** 2026-05-18

**Authors:** Qian Jun Beh, Moritz Straeter, Zeen Sun, Leong Chuan Kwek, Yuancheng Zhan

**Affiliations:** 1 Centre for Quantum Technologies (CQT), National University of Singapore, Singapore 117543, Singapore; qjbeh@u.nus.edu (Q.J.B.); moritz.straeter@u.nus.edu (M.S.); zeen_sun@u.nus.edu (Z.S.); 2 School of Electrical and Electronic Engineering (EEE), Nanyang Technological University, Singapore 639798, Singapore; 3 National Institute of Education (NIE), Nanyang Technological University, Singapore 637616, Singapore; 4 MajuLab, CNRS-UNS-NUS-NTU International Joint Research Unit, Singapore 117543, Singapore

**Keywords:** quantum information, quantum cloning, quantum autoencoding

## Abstract

Quantum cloning machines are essential in quantum information processing, finding applications in areas such as quantum communication and cryptographic protocols. However, the fidelity of universal quantum cloning machines diminishes as the dimension of the Hilbert space increases, resulting in significantly lower efficiency when cloning high-dimensional quantum states compared to qubits. In this study, we introduce a Hybrid Quantum Autocloning Machine (HQAM) that combines quantum autoencoding with universal quantum cloning. The core concept involves compressing a high-dimensional quantum state into a lower-dimensional effective subspace through a quantum autoencoder, conducting the cloning process within this reduced subspace, and then reconstructing the state in the original Hilbert space. Our results show that, for input states with a strong overlap with the effective qubit subspace, the HQAM achieves cloning fidelities exceeding the benchmark fidelity of direct qutrit universal cloning and approaching the optimal qubit cloning limit, while maintaining robustness under noise. These findings demonstrate that compression-assisted cloning provides a practical strategy for improving cloning performance in high-dimensional quantum systems and may enable more efficient quantum information processing protocols.

## 1. Introduction

Quantum information processing unlocks fundamentally new capabilities for computation [1,2], communication [3,4], and simulation [5,6,7,8]. However, the practical implementation of quantum technologies is hindered by limited quantum resources. In near-term platforms [9,10,11,12], there is a scarcity of quantum memory, coherence times are finite, and manipulating high-dimensional quantum states often requires significant experimental and computational resources. These challenges highlight the need for protocols that leverage the intrinsic structure of quantum states to reduce the effective dimensionality of the information processed. A notable example is quantum cloning; while the no-cloning theorem prohibits the perfect duplication of arbitrary unknown quantum states [13], approximate cloning is permissible and plays a crucial role in quantum information theory. Universal quantum cloning machines (UQCMs) can produce optimal approximate copies of any state [14], but the fidelity of cloning diminishes as the dimension of the Hilbert space increases. This means that cloning higher-dimensional states, like qutrits, is inherently less accurate than cloning qubits. This observation suggests that if a high-dimensional state primarily exists within a lower-dimensional effective subspace, cloning within that reduced subspace could offer a more resource-efficient approach.

Quantum autoencoders (QAEs) [15,16] offer a natural framework for such dimensionality reduction. As the quantum analogue of classical autoencoders, QAEs compress quantum states into lower-dimensional latent spaces while preserving the information required for faithful reconstruction [15]. This approach is particularly effective when the relevant states occupy only a restricted region of the Hilbert space. Previous work has demonstrated both theoretical and experimental implementations of quantum compression, including photonic schemes that map qutrit states into effective qubit subspaces [17]. From a structural viewpoint, the optimal compression of an ensemble $\left\{p_{i},|\psi_{i}\rangle \right\}$ is the principal component projector of its average state $\bar{\rho }$—that is, quantum principal component analysis (quantum PCA) on the ensemble. In a fully classical simulation, this projector can be computed in closed form by diagonalising the empirical $\bar{\rho }$. In a quantum setting, however, $\bar{\rho }$ is not available, and obtaining it would require full state tomography of the ensemble. Quantum autoencoders provide a sample-based, tomography-free realisation of the same projector by training a parametrised unitary on per-state measurements alone [15,16,18], and are therefore the natural method when the latent subspace is not known a priori. Meanwhile, approximate quantum cloning has been extensively studied in both theory and experiments. The Bužek–Hillery UQCM provides the optimal symmetric 1→2 cloning protocol for qubits [19]. Despite these advances, quantum autoencoding and quantum cloning have largely been studied independently, and the potential benefits of combining state compression with quantum cloning remain largely unexplored.

In this work, we investigate a compression-assisted cloning strategy for high-dimensional quantum states that possess significant overlap with a lower-dimensional subspace. Instead of directly applying a *d*-dimensional cloning protocol, the input state is first compressed into an effective qubit subspace, cloning is performed within this reduced space, and the resulting states are subsequently reconstructed in the original Hilbert space. To realise this idea, we introduce a *Hybrid Quantum Autocloning Machine* (HQAM) that integrates a quantum autoencoder with the Bužek–Hillery UQCM. In this protocol, the encoder compresses the input qutrit state into a qubit representation while suppressing the population in an auxiliary junk mode. Cloning is then performed within the encoded qubit subspace, after which the cloned states are decoded back into the qutrit space.

We analyse this protocol using both analytical modelling and numerical optimisation. Our results show that on Haar-scrambled qutrit ensembles—in which the latent two-dimensional subspace *V* is unknown to the encoder—the trained autoencoder converges to the analytical quantum-PCA optimum within statistical precision. The resulting cloning fidelity exceeds the universal quitrit cloning bound F=3/4 when the overlap with *V* is sufficiently large ($\chi \geq 3/\sqrt{10}$) and approaches the qubit cloning limit F=5/6 as χ→1. By contrast, a fixed canonical projection—equivalent to assuming the latent subspace is known and aligned with the lab basis—cannot solve the problem when the subspace is unknown; its mean fidelity sits at the Haar-random scramble limit 5/9, independent of χ. The protocol degrades gracefully under leakage noise that drives states away from the effective qubit subspace. These results demonstrate that compression-assisted cloning provides a subspace-aware strategy for improving cloning performance in high-dimensional quantum systems whose latent structure is not known a priori and offer a pathway towards practical implementations of hybrid quantum cloning protocols.

## 2. Hybrid Quantum Autocloning Machine

We focus here on the theoretical and numerical HQAM framework. An illustrative photonic realisation of the encoding process is in Appendix A.

In quantum cloning, the no-cloning theorem states that no physical operation can perfectly copy an arbitrary unknown quantum state [13]. Nevertheless, approximate cloning is possible, and optimal approximate cloning protocols have been extensively studied. The Bužek–Hillery universal quantum cloning machine generates optimal symmetric copies of arbitrary qubit states. For a general qubit input state |ψ〉=α|0〉+β|1〉, the cloning transformation can be written as

(1) $$\begin{array}{c} |\psi \rangle |R\rangle |M\rangle \to \sqrt{\frac{2}{3}}|\psi \rangle |\psi \rangle |\psi^{⊥}\rangle -\sqrt{\frac{1}{6}}\left[|\psi \rangle |\psi^{⊥}\rangle +|\psi^{⊥}\rangle |\psi \rangle \right]|\psi \rangle . \end{array}$$

The performance of a cloning protocol is characterised by the cloning fidelity *F*, which measures the similarity between the cloned state and the original input. For universal symmetric N→M cloning of a *d*-dimensional quantum system, the optimal fidelity is given by [14]

(2) $$F_{N\to M}\left(d\right)=\frac{N}{M}+\frac{\left(M-N)(N+1\right)}{M(N+d)}.$$

Since the optimal fidelity decreases as the Hilbert-space dimension *d* increases, cloning higher-dimensional quantum states is intrinsically less accurate than cloning lower-dimensional states. This limitation motivates the search for strategies that can exploit the structure within the state space to improve cloning performance.

Quantum autoencoders provide a natural framework for such dimensionality reduction. A quantum autoencoder compresses quantum states into lower-dimensional representations while preserving the information required for reconstruction. In a QAE, an encoder maps an input state in a high-dimensional Hilbert space into a lower-dimensional latent subspace, and a decoder subsequently reconstructs the original state from the compressed representation. Formally, the encoding process can be written as

$${|\psi \rangle }_{\mathrm{in}}\to U_{e}{|\psi \rangle }_{\mathrm{in}}={|\psi \rangle }_{\mathrm{enc}},$$

where $U_{e}$ is a unitary encoder, and the encoded state occupies an effective subspace of the original Hilbert space. The decoder reconstructs the input state through the inverse transformation $U_{e}^{†}$. If the relevant quantum states predominantly occupy a lower-dimensional subspace, compressing the state prior to cloning may, therefore, provide a more resource-efficient strategy. This observation forms the basis of the compression-assisted cloning approach investigated in this work.

### 2.1. Analytical Model

Consider a general normalised qutrit state ${|\psi \rangle }_{\mathrm{in}}=c_{0}|0\rangle +c_{1}|1\rangle +c_{2}|2\rangle$, with ${\left|c_{0}\right|}^{2}+{\left|c_{1}\right|}^{2}+{\left|c_{2}\right|}^{2}=1$ and $c_{0},c_{1},c_{2}\in C$. The quantum autoencoder first applies an encoding unitary, giving $U_{e}{|\psi \rangle }_{\mathrm{in}}$, and then projects into a two-dimensional qutrit subspace, yielding the normalised logical qubit state

(3) $$\begin{array}{c} |\psi_{\mathrm{enc}}\rangle =\frac{\hat{\Pi }U_{e}{|\psi \rangle }_{\mathrm{in}}}{\sqrt{1-\varepsilon }}, \end{array}$$

where $\hat{\Pi }=|1\rangle \langle 1|+|2\rangle \langle 2|$ and ε is the compression error, given by

(4) $$\begin{array}{c} \varepsilon =\mathrm{tr}\left[\left(I-\hat{\Pi }\right)U_{e}{|\psi \rangle }_{\mathrm{in}}{\langle \psi |}_{\mathrm{in}}U_{e}^{†}\right]. \end{array}$$

The projection is successful with probability 1−ε. Applying the Bužek–Hillery qubit quantum cloning machine to the encoded logical qubit state $|\psi_{\mathrm{enc}}\rangle$ yields

(5) $$\begin{array}{c} \rho_{\mathrm{enc}}^{\left(A\right)}=\rho_{\mathrm{enc}}^{\left(B\right)}=\frac{5}{6}|\psi_{\mathrm{enc}}\rangle \langle \psi_{\mathrm{enc}}|+\frac{1}{6}|\psi_{\mathrm{enc}}^{⊥}\rangle \langle \psi_{\mathrm{enc}}^{⊥}|, \end{array}$$

where $|\psi_{\mathrm{enc}}^{⊥}\rangle$ is the state in the encoded two-dimensional subspace orthogonal to $|\psi_{\mathrm{enc}}\rangle$. Decoding with $U_{e}^{†}$ gives

(6) $$\begin{array}{c} \rho_{\mathrm{out}}^{\left(A\right)}=\rho_{\mathrm{out}}^{\left(B\right)}=U_{e}^{†}\left(\frac{5}{6}|\psi_{\mathrm{enc}}\rangle \langle \psi_{\mathrm{enc}}|+\frac{1}{6}|\psi_{\mathrm{enc}}^{⊥}\rangle \langle \psi_{\mathrm{enc}}^{⊥}|\right)U_{e}. \end{array}$$

The one-copy fidelity *F* with the input state is therefore

(7) $$\begin{array}{cc} \;\;\;\;\;\;\;\;\;\;\;\;\;\;\;F\; & ={\langle \psi |}_{\mathrm{in}}U_{e}^{†}\left(\frac{5}{6}|\psi_{\mathrm{enc}}\rangle \langle \psi_{\mathrm{enc}}|+\frac{1}{6}|\psi_{\mathrm{enc}}^{⊥}\rangle \langle \psi_{\mathrm{enc}}^{⊥}|\right)U_{e}{|\psi \rangle }_{\mathrm{in}} \end{array}$$

(8) $$\begin{array}{cc} \; & =\frac{5}{6}{\left|{\langle \psi |}_{\mathrm{in}}U_{e}^{†}|\psi_{\mathrm{enc}}\rangle \right|}^{2}+\frac{1}{6}{\left|{\langle \psi |}_{\mathrm{in}}U_{e}^{†}|\psi_{\mathrm{enc}}^{⊥}\rangle \right|}^{2}. \end{array}$$

Since $|\psi_{\mathrm{enc}}\rangle$ is proportional to $\hat{\Pi }U_{e}{|\psi \rangle }_{\mathrm{in}}$, we have

(9) $$\begin{array}{c} {\left|{\langle \psi |}_{\mathrm{in}}U_{e}^{†}|\psi_{\mathrm{enc}}\rangle \right|}^{2}=1-\varepsilon . \end{array}$$

Moreover, because $|\psi_{\mathrm{enc}}^{⊥}\rangle$ lies in the range of $\hat{\Pi }$ and is orthogonal to $|\psi_{\mathrm{enc}}\rangle$,

(10) $$\begin{array}{c} {\langle \psi |}_{\mathrm{in}}U_{e}^{†}|\psi_{\mathrm{enc}}^{⊥}\rangle =0. \end{array}$$

Hence

(11) $$\begin{array}{c} F=\frac{5}{6}\left(1-\varepsilon \right). \end{array}$$

The HQAM produces copies with higher fidelity than direct universal qutrit cloning if

(12) $$\begin{array}{c} \frac{5}{6}\left(1-\varepsilon \right)>\frac{3}{4}\;\Rightarrow \;\varepsilon <\frac{1}{10}. \end{array}$$

For an ensemble of qutrit quantum states $E=\{p_{i},|\psi_{i}\rangle \}$ with average state

(13) $$\begin{array}{c} \bar{\rho }=\sum_{i}p_{i}|\psi_{i}\rangle \langle \psi_{i}|, \end{array}$$

the average compression error $\bar{\varepsilon }$ is given by

(14) $$\begin{array}{c} \bar{\varepsilon }=1-\mathrm{tr}\left[\hat{\Pi }U_{e}\bar{\rho }U_{e}^{†}\right]. \end{array}$$

This quantity is minimised when $U_{e}$ diagonalises $\bar{\rho }$ and maps the two eigenvectors with the largest eigenvalues to |1〉 and |2〉. For an average state $\bar{\rho }$ with eigenvalues $\lambda_{1}\geq \lambda_{2}\geq \lambda_{3}$, the minimum average compression error is

(15) $$\begin{array}{c} {\bar{\varepsilon }}_{\min}=\lambda_{3}. \end{array}$$

The corresponding maximum average cloning fidelity is

(16) $$\begin{array}{c} {\bar{F}}_{\max}=\frac{5}{6}\left(\lambda_{1}+\lambda_{2}\right)=\frac{5}{6}\left(1-\lambda_{3}\right)=\frac{5}{6}\left(1-{\bar{\varepsilon }}_{\min}\right). \end{array}$$

In the limit of perfect compressibility of the ensemble ($\bar{\varepsilon }=0$), the qubit cloning fidelity F=5/6 is recovered, as expected.

Equation (16) characterises the optimal encoder for an ensemble $\left\{p_{i},|\psi_{i}\rangle \right\}$: the unitary $U_{e}$ diagonalises the average state $\bar{\rho }$ and maps its two leading eigenvectors onto the qubit subspace {|1〉,|2〉}, while the eigenvector with the smallest eigenvalue is mapped to the junk mode |0〉. This is the principal component projector of $\bar{\rho }$, i.e., quantum principal component analysis of the ensemble. The numerical implementation in Section 2.2 provides a sample-based realisation of this projector that does not require explicit access to $\bar{\rho }$.

### 2.2. Numerical Implementation

**Figure 1:**
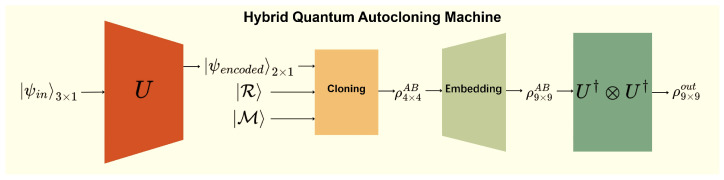
Illustration of the hybrid autocloning machine. The input is a qutrit state, the encoder outputs an encoded qubit state in the effective two-dimensional subspace, and $U^{†}⊗U^{†}$ denotes the decoder. The labels “3×1” and “2×1” denote qutrit and qubit column vectors, respectively.

To complement the illustrative photonic discussion in Appendix A, we numerically evaluate the performance of the Hybrid Quantum Autocloning Machine. As illustrated in Figure 1, the protocol consists of three stages: the input qutrit state is first compressed into an effective qubit subspace by an optimised encoder, the encoded state is then cloned using the Bužek–Hillery universal quantum cloning machine, and the resulting two-clone state is finally decoded back into the qutrit space. The performance of the HQAM is quantified by the fidelity between the decoded output state and the original input state.

We model the realistic setting in which the encoder receives states drawn from a structured ensemble whose latent two-dimensional subspace is unknown a priori. Datasets are generated in two steps: (i) draw a state |φ〉 in canonical coordinates with the |0〉 component fixed in magnitude and a uniformly random phase and the (|1〉,|2〉) sampled uniformly on the χ-radius sphere within span{|1〉,|2〉},

(17) $$\begin{array}{c} |\varphi \rangle =c_{0}|0\rangle +c_{1}|1\rangle +c_{2}|2\rangle ,\;|c_{0}{|}^{2}=1-\chi^{2},\;|c_{1}{|}^{2}+{\left|c_{2}\right|}^{2}=\chi^{2}, \end{array}$$

so that every state has overlap with span{|1〉,|2〉} exactly equalt to χ; (ii) apply a fixed Haar-random unitary $U_{\mathrm{scramble}}\in U\left(3\right)$ so that the actual input state is

(18) $$\begin{array}{c} |\psi_{\mathrm{in}}\rangle =U_{\mathrm{scramble}}\;|\varphi \rangle . \end{array}$$

The resulting ensemble has fixed overlap χ wit hthe rotated subspace $V=U_{\mathrm{scramble}}\cdot \mathrm{span}\left\{|1\rangle ,|2\rangle \right\}$ (a fully two-dimensional plane in $C^{3}$; the parameter χ controls how concentrated the data is within *V*, not the dimensionality of *V* itself). The encoder receives $|\psi_{\mathrm{in}}\rangle$ without any access to $U_{\mathrm{scramble}}$ or to the empirical $\bar{\rho }$. Seven values χ∈{0.85,0.875,0.90,0.925,0.95,0.975,0.99} are considered, and ten independent draws of $U_{\mathrm{scramble}}$ are used to average over the choice of latent subspace. Each $\left(\chi ,U_{\mathrm{scramble}}\right)$ pair contributes a training set of 2000 states and a held-out test set of 500 states. Details of the state generation procedure are provided in Appendix C. Because every state has overlap exactly χ with *V* and is uniform within *V*, the average state has eigenvalues $\left(\chi^{2}/2,\;\chi^{2}/2,\;1-\chi^{2}\right)$ with eigenvectors aligned with *V* and $V^{⊥}$. For $\chi >\sqrt{2/3}$, which covers the entire range considered here, the smallest eigenvalue is $\lambda_{3}=1-\chi^{2}$, and Equation (16) reduces to

(19) $${\bar{F}}_{\max}\left(\chi \right)\;=\;\frac{5}{6}\;\chi^{2}.$$

This is the direct counterpart of Equation (16) for the structured ensembles benchmarked in Section 3.1; the empirical HQAM fidelities reported in Table 1 agree with Equation (19) to within sample noise (≲$5\times 10^{-4}$). The encoder is parametrised as

(20) $$\begin{array}{c} U_{e}=e^{iH},\;H=\sum_{j=1}^{8}w_{j}\sigma_{j}, \end{array}$$

where $\sigma_{j}$ are the Gell–Mann matrices and $w_{j}$ are trainable parameters. The choice of a parametric encoder optimised from per-state measurements is what makes the protocol applicable in a quantum setting where the ensemble is provided as a stream of physical states rather than classical descriptions. As shown in Section 2.1, the optimal encoder for a given ensemble is the principal component projector of $\bar{\rho }$. In a fully classical simulation, this projector can be computed in closed form by diagonalising the empirical $\bar{\rho }$. In a quantum setting, however, $\bar{\rho }$ is not directly available; obtaining it would require full state tomography of the ensemble. The autoencoder side-steps this by training $U_{e}$ on per-state measurements of the junk mode population alone, providing a sample-based, tomography-free realisation of quantum PCA on the input ensemble [15,16,17]. We verify in Section 3.1 that the trained encoder converges to the analytical optimum of Equation (16) within statistical precision. The encoded state is obtained as

(21) $$\begin{array}{c} |\psi_{\mathrm{encoded}}\rangle =U_{e}|\psi_{\mathrm{in}}\rangle ,\;L_{\mathrm{junk}}={\left|\langle 0|\psi_{\mathrm{encoded}}\rangle \right|}^{2}. \end{array}$$

Here $|\psi_{\mathrm{encoded}}\rangle$ is still formally a qutrit-state vector in the three-dimensional Hilbert space. When the junk mode population is suppressed, the relevant information is concentrated in the effective two-dimensional subspace, and only this qubit component is passed to the cloning stage.

The encoded state is then processed by the Bužek–Hillery UQCM. In numerical form, the cloning stage is described by

(22) $$\begin{array}{c} {|\psi_{\mathrm{encoded}}\rangle }_{2\times 1}\overset{\mathrm{cloning}}{\to }{|\psi_{\mathrm{cloned}}\rangle }_{8\times 1}\to \rho_{8\times 8}^{\mathrm{cloned}}\overset{{\mathrm{Tr}}_{M}\left[\rho \right]}{\to }\rho_{4\times 4}^{AB}, \end{array}$$

where *A* and *B* denote the two clone subsystems, and the machine degree of freedom has been traced out. The subscript “2×1” here denotes the two-component column vector associated with the effective qubit subspace, not the full encoded qutrit representation. To decode the cloned state, we take the decoder unitary to be the adjoint of the encoder,

(23) $$\begin{array}{c} U_{d}=U_{e}^{†}⊗U_{e}^{†}, \end{array}$$

since the decoding acts on the tensor product space of the two clones. As the cloned state $\rho^{AB}$ lies in a two-qubit space while the decoder acts in the two-qutrit space, we embed $\rho_{4\times 4}^{AB}$ into the 9×9 qutrit tensor space using the isometry

(24) $$\begin{array}{c} I_{3\times 2}=\left[\begin{array}{cc} 0 & 0\\ 1 & 0\\ 0 & 1 \end{array}\right],\;I_{3\times 2}^{†}I_{3\times 2}=I_{2\times 2}. \end{array}$$

The embedded cloned state is therefore

(25) $$\begin{array}{c} \rho_{9\times 9}^{AB}=\left(I_{3\times 2}⊗I_{3\times 2}\right)\rho_{4\times 4}^{AB}{\left(I_{3\times 2}⊗I_{3\times 2}\right)}^{†}, \end{array}$$

which is then decoded as

(26) $$\rho_{\mathrm{decoded}}=U_{d}\;\rho_{9\times 9}^{AB}\;U_{d}^{†}.$$

The state $\rho_{\mathrm{decoded}}$ in Equation (26) is the full jointly decoded two-qutrit state. The single-clone fidelities reported in the numerical results are computed from its one-clone marginals,

(27) $$\begin{array}{cccc} \rho_{\mathrm{decoded}}^{A} & ={\mathrm{Tr}}_{B}\;\left[\rho_{\mathrm{decoded}}\right],\; & \rho_{\mathrm{decoded}}^{B} & ={\mathrm{Tr}}_{A}\;\left[\rho_{\mathrm{decoded}}\right], \end{array}$$

with

(28) $$\begin{array}{cccc} F_{A} & =\langle \psi_{\mathrm{in}}|\rho_{\mathrm{decoded}}^{A}|\psi_{\mathrm{in}}\rangle ,\; & F_{B} & =\langle \psi_{\mathrm{in}}|\rho_{\mathrm{decoded}}^{B}|\psi_{\mathrm{in}}\rangle . \end{array}$$

Equivalently, because the decoder is local on the two clone systems, tracing out one subsystem after applying $U_{e}^{†}⊗U_{e}^{†}$ gives the same one-clone marginal as decoding the corresponding reduced clone state separately. The separate reduced-clone implementation therefore evaluates the same $F_{A}$ and $F_{B}$ as the marginal evaluation of the full two-qutrit decoded state, although it does not store the full joint correlations.

The encoder parameters are optimised by minimising the total loss

(29) $$L\;=\;L_{\mathrm{cloning}}\;+\;\beta \;L_{\mathrm{junk}},$$

where $L_{\mathrm{cloning}}=1-F_{A}-F_{B}+{\left(F_{A}-F_{B}\right)}^{2}$ and $L_{\mathrm{junk}}={\left|\langle 0|\psi_{\mathrm{encoded}}\rangle \right|}^{2}$, $F_{A}$ and $F_{B}$ denote the fidelities of the two decoded clones, and the penalty term ${\left(F_{A}-F_{B}\right)}^{2}$ enforces approximate symmetry between them. The parameter β controls the relative weight of the junk mode suppression term. For the symmetric Bužek–Hillery cloner of Section 2.1, the symmetry penalty ${\left(F_{A}-F_{B}\right)}^{2}$ vanishes identically, $F_{A}=F_{B}$, and so contributes nothing to the gradient; it is retained as a guard for non-ideal or asymmetric cloning channels.

We analyse the algebraic structure of the loss in the ideal pipeline. The cloning step in Section 2.1 is a Bužek–Hillery universal cloner; by universality, the per-state single-clone fidelities depend on the input only through its overlap χ(ψ) with the encoder’s qubit subspace, $F_{A}\left(\psi \right)=F_{B}\left(\psi \right)=\frac{5}{6}\;\chi^{2}\left(\psi \right)$. Substituting into the loss definitions gives $L_{\mathrm{cloning}}\left(\psi \right)=1-\frac{5}{3}\chi^{2}\left(\psi \right)$ and $L_{\mathrm{junk}}\left(\psi \right)=1-\chi^{2}\left(\psi \right)$, so the total loss reduces to

(30) $$L\left(\psi \right)\;=\;\left(1+\beta \right)\;-\;\left(\frac{5}{3}+\beta \right)\chi^{2}\left(\psi \right),$$

an affine function of $\chi^{2}\left(\psi \right)$ alone. Averaging over the training ensemble, minimising 〈L〉 is therefore equivalent to maximising $\langle \chi^{2}\rangle$, which is exactly the criterion that defines the analytical optimum (16). The loss is structurally consistent with the quantum-PCA solution of Section 2.1: any choice of β>0 drives the encoder to the same minimiser, and the role of β is only to rescale the gradient magnitude during training. The cloning loss term is retained because it makes the operational objective (cloning fidelity) explicit in the loss and because the reduction (30) relies on universality of the ideal cloner: as soon as the cloning channel deviates from the Bužek–Hillery form (for example, under cloning-stage noise), $F_{A}$ ceases to be a function of $\chi^{2}$ alone and the two loss terms become structurally distinct.

All simulations were implemented in Python 3.10 on an RTX 4090 GPU. The trainable parameters are the coefficients $w=(w_{1},\ldots ,w_{8})$ appearing in the encoder Hamiltonian $H=\sum_{j=1}^{8}w_{j}\sigma_{j}$. These parameters are trained once for an ensemble of qutrit states with a prescribed overlap χ and then kept fixed. Accordingly, the HQAM is designed for structured families of input states with prior subspace information, rather than arbitrary unknown qutrit states.

For reproducibility and held-out evaluation, every $\left(\chi ,U_{\mathrm{scramble}}\right)$ combination is checked in as separate pseudo-training and pseudo-test files. For the main setting β=8.0, η=0.01, training is repeated for five random weight initialisations (seeds 0–4) for each of ten independent draws of $U_{\mathrm{scramble}}$ (seeds 0–9), giving 50 trained encoders per χ. Each run uses 200 training epochs, all 2000 training states, and all 500 held-out test states. Aggregated statistics are reported in Table 1. The standard deviation captures both seed-to-seed variation in the trained weights and scramble-to-scramble variation in the latent subspace.

The parameters were optimised using gradient descent to minimise the total loss function defined in Equation (29). At iteration *k*, the parameter vector is updated according to

(31) $$w_{k+1}=w_{k}-\eta \;\nabla_{w}L\left(w_{k}\right),$$

where η denotes the learning rate controlling the step size of the optimisation. All trained encoders reported below use η=0.01 and β=8, for which training converges to the analytical optimum within ∼25 epochs; smaller learning rates (e.g., η=0.001) reach the same optimum but require substantially more epochs.

## 3. Results

In this section, we present the performance of the hybrid autocloning machine with the corresponding code available in [20].

We first benchmark the cloning fidelity achieved by the HQAM against the theoretical limits of universal cloning on Haar-scrambled structured ensembles, then investigate the robustness of the protocol under leakage noise that drives the state away from the effective qubit subspace.

### 3.1. Cloning Fidelity Benchmark

We benchmark the cloning performance of the HQAM on the Haar-scrambled ensembles described in Section 2.2, in which the latent two-dimensional subspace $V=U_{\mathrm{scramble}}\cdot \mathrm{span}\left\{|1\rangle ,|2\rangle \right\}$ is unknown to the encoder. The reference values are the universal symmetric 1→2 cloning fidelities of Equation (2): F=5/6 for qubits and F=3/4 for qutrits. The analytical maximum achievable on a structured ensemble with smallest eigenvalue $\lambda_{3}$ of $\bar{\rho }$ is ${\bar{F}}_{\max}=\left(5/6\right)\left(1-\lambda_{3}\right)$, realised by the quantum-PCA encoder of Section 2.1.

The benchmark comparison in this work is intentionally restricted to universal cloning benchmarks. In particular, the relevant direct qutrit benchmark is the universal qutrit cloning fidelity F=3/4. For the structured ensembles considered here, the HQAM exceeds this direct universal qutrit cloning benchmark when the input states have sufficiently large overlap with the effective qubit subspace. This result should be interpreted as improved performance relative to universal qutrit cloning on structured input families, rather than as a claim of optimality among all state-dependent qutrit cloners.

**Figure 2:**
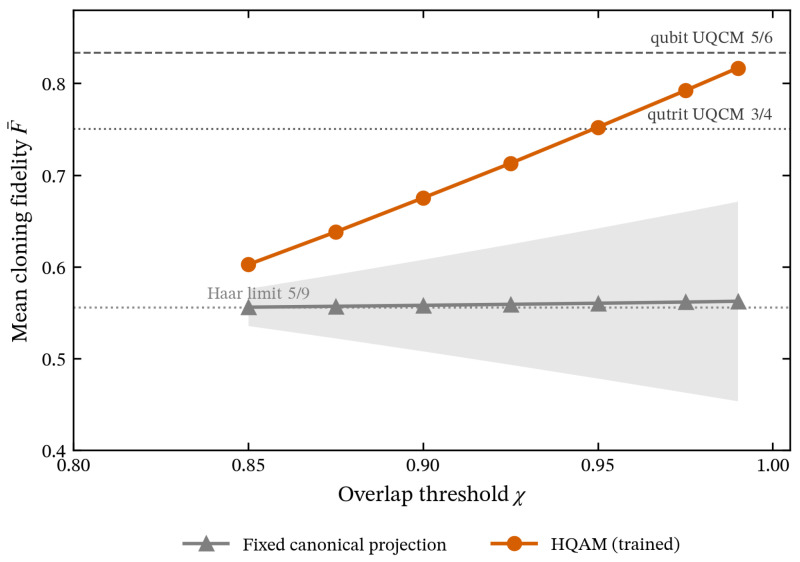
Cloning fidelity of HQAM versus the fixed canonical projection on Haar-scrambled qutrit ensembles, as a function of the overlap χ. Each input state exactly overlaps χ with the latent subspace *V*. Each point aggregates 10 Haar-random draws of $U_{\mathrm{scramble}}$; HQAM additionally averages over 5 random weight initialisations per scramble. Shaded bands indicate ±std across scramble seeds; the HQAM band is smaller than the markers. The trained HQAM encoder (orange) saturates the analytical maximum ${\bar{F}}_{\max}\left(\chi \right)=\left(5/6\right)\chi^{2}$ of Equation (19)—the quantum-PCA optimum on the empirical $\bar{\rho }$—and exceeds the universal qutrit cloning bound F=3/4 for $\chi >\chi_{\mathrm{crit}}=3/\sqrt{10}\approx 0.9487$, approaching the qubit cloning limit F=5/6 as χ→1. The fixed canonical projection (gray) lacks access to the unknown latent *V*; its mean fidelity sits at the Haar-random scramble limit 5/9≈0.556, independent of χ.

**Figure A3:**
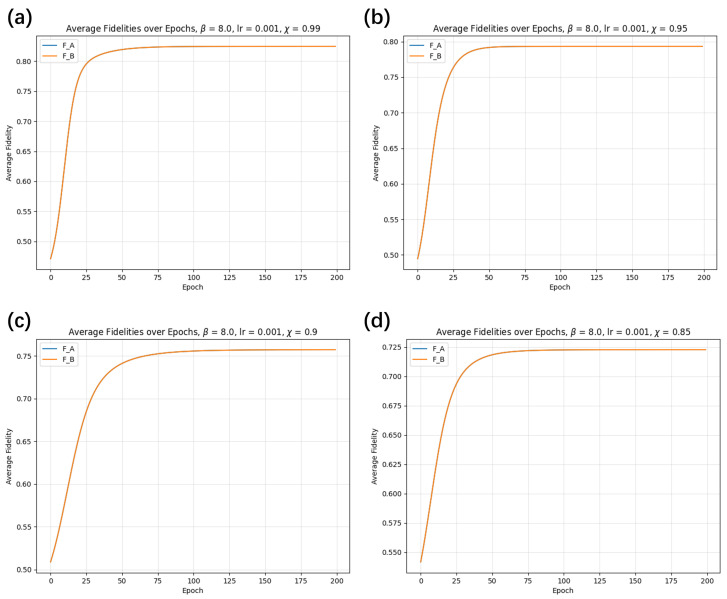
Plots for fidelities of both copies, $F_{A}$ and $F_{B}$, for different χ at β=8.0 and η=0.001. (**a**) χ=0.99. (**b**) χ=0.95. (**c**) χ=0.90. (**d**) χ=0.85. The $F_{A}$ and $F_{B}$ are overlap, which means they are identical, aligning with the theory.

Figure 2 reports the held-out cloning fidelity as a function of χ for two encoders: HQAM (trained on $|\psi_{\mathrm{in}}\rangle$ via the procedure of Section 2.2) and a fixed canonical projection (the trivial isometry that maps $|\psi_{\mathrm{in}}\rangle$ onto span{|1〉,|2〉} of the lab basis without any learning). Three observations can be made:

*(i) HQAM saturates the analytical maximum.* For every χ, the trained encoder agrees with the analytical prediction ${\bar{F}}_{\max}\left(\chi \right)=\left(5/6\right)\chi^{2}$ from Equation (19) to within $∼5\times 10^{-4}$ (Table 1), and the seed-to-seed standard deviation is below $2\times 10^{-4}$. We additionally verified numerically that the analytical quantum-PCA encoder—obtained by diagonalising the empirical $\bar{\rho }$ on the training set—agrees with the trained HQAM to within $\max|{\bar{F}}_{\mathrm{HQAM}}-{\bar{F}}_{\mathrm{PCA}}|\;<2\;\times \;10^{-4}$ across all $\left(\chi ,U_{\mathrm{scramble}}\right)$ combinations, consistent with the analytical prediction of Section 2.1: the autoencoder converges to the principal component projector of $\bar{\rho }$ from per-state measurements alone. The fidelities of the two clones coincide ($F_{A}=F_{B}$; see Figure A3), confirming the symmetry expected from the Bužek–Hillery protocol.

*(ii) HQAM exceeds the universal qutrit cloning bound for sufficient χ.* The trained encoder approaches the qubit cloning limit 5/6 as χ→1 ($\bar{F}=0.817$ at χ=0.99). The crossover with the universal qutrit bound F=3/4 follows directly from Equation (19): setting $\frac{5}{6}\chi^{2}=3/4$ gives

(32) $$\chi_{\mathrm{crit}}\;=\;\sqrt{\frac{9}{10}}\;=\;\frac{3}{\sqrt{10}}\;\approx \;0.9487.$$

For $\chi >\chi_{\mathrm{crit}}$, the structured ensemble is sufficiently compressible that HQAM exceeds direct universal qutrit cloning; for $\chi <\chi_{\mathrm{crit}}$, even the analytical optimum ${\bar{F}}_{\max}$ remains below 3/4, so subspace exploitation cannot beat the universal qutrit cloner. The empirical crossover between χ=0.925 ($\bar{F}=0.713$) and χ=0.95 ($\bar{F}=0.752$) in Figure 2 matches this bound.

*(iii) The fixed canonical projection fails on scrambled data.* Without access to $U_{\mathrm{scramble}}$, projecting onto span{|1〉,|2〉} of the lab basis achieves $\bar{F}\approx 0.556$ across all χ values, with a 10-scramble band whose width grows from ±0.02 at χ=0.85 to ±0.11 at χ=0.99. This is consistent with the analytically predicted Haar-random scramble limit 5/9≈0.556: in expectation over Haar-random $U_{\mathrm{scramble}}$, the per-state cloning fidelity reduces to $\left(5/6\right)\langle \chi_{\mathrm{lab}}^{2}\rangle =\left(5/6\right)\cdot \left(2/3\right)=5/9$, independent of χ. The gap to HQAM grows from 0.05 at χ=0.85 to 0.26 at χ=0.99, confirming that learning is required when the latent subspace is unknown; a fixed canonical isometry suffices only when the input distribution is hand-aligned with the lab basis.

### 3.2. Robustness Under Leakage Noise

The leakage noise analysis below uses the canonical-aligned ensemble (no Haar scrambling) in order to isolate the effect of leakage on the cloning fidelity from the subspace recovery question of Section 3.1. The noise strength ϵ in this section therefore plays the role of a channel parameter rather than the structural overlap threshold χ of Section 2.2. The overlap of the input qutrit with the effective qubit subspace, denoted by χ, can be interpreted as the action of a noise channel that drives the state away from the ideal two-dimensional encoding space. From the perspective of the encoded qubit, this behaviour resembles the effect of a depolarising process. For reference, the standard depolarising channel acting on a qubit density matrix ρ is given by

(33) $$\rho \to \left(1-\epsilon \right)\rho +\epsilon \frac{I}{2},$$

where ϵ denotes the noise probability. A general derivation of depolarising channels for *d*-dimensional systems and a special case d=2 are provided in Appendix E.1 and Appendix E.2, respectively.

In the present work, the leakage from the qubit subspace is modelled as a channel that transfers population from the encoded qubit subspace into the fixed qutrit state |0〉. The corresponding mapping can be written as

(34) ρ→(1−ϵ)ρ+ϵ|0〉〈0|,

where ϵ:=1−χ and the effective qubit subspace is spanned by {|1〉,|2〉}. A proof that this mapping constitutes a valid, completely positive, and trace-preserving (CPTP) operation is given in Appendix E.3.

**Figure 3:**
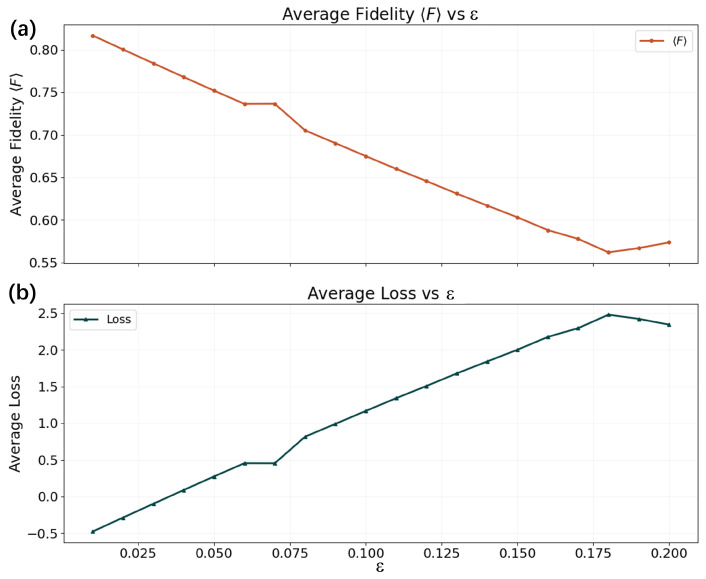
Performance of the HQAM with the leakage channel, where ϵ∈[0.01,0.20]. (**a**) Average fidelity 〈F〉 versus ϵ. (**b**) Average loss versus ϵ. The values for both fidelity and loss are taken at the plateau during training for each ϵ.

**Figure A4:**
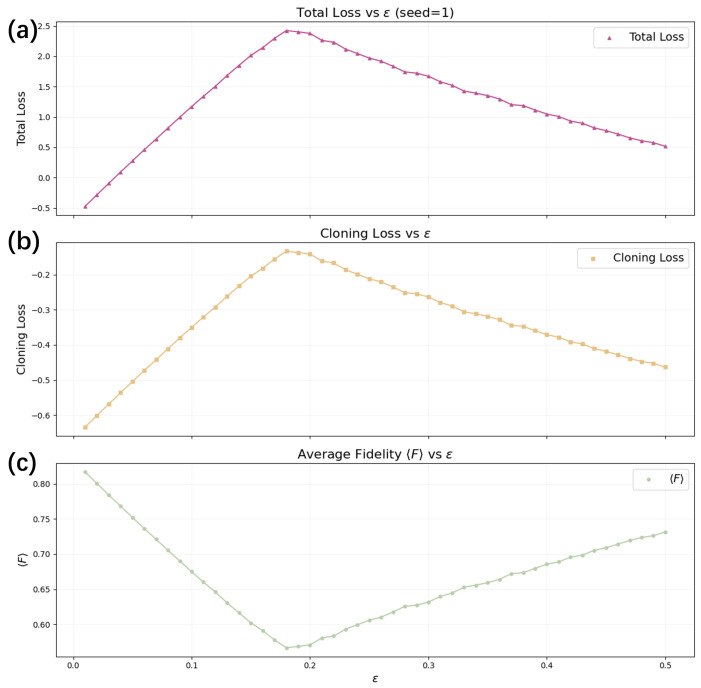
Performance of the HQAM under the leakage channel for ϵ∈[0.01,0.50] in a representative fixed-seed run. (**a**) Total loss as a function of ϵ, showing the overall optimisation objective used in training. (**b**) Cloning loss as a function of ϵ, isolating the cloning-related contribution to the objective. (**c**) Average fidelity 〈F〉 as a function of ϵ, showing the non-monotonic dependence of cloning performance on the leakage strength over this interval.

We evaluate the robustness of the HQAM under this leakage model by training the protocol on noisy input states with ϵ∈[0.01,0.20]. The corresponding results are shown in Figure 3. As expected, the average cloning fidelity initially decreases as the noise level increases, reflecting the degradation of information encoded in the qubit subspace. However, a non-monotonic behaviour appears when ϵ approaches the upper end of this interval. This effect is analysed more systematically by extending the simulations to ϵ∈[0.18,0.50], as shown in Figure A4.

The observed behaviour can be understood analytically by considering the linearity of the HQAM pipeline. The full protocol consists of a sequence of operations—encoding, cloning, embedding, partial tracing, and decoding—each of which is a valid CPTP map. Consequently, the overall HQAM transformation σ(·) is also linear. Applying this map to the noisy state in Equation (34) gives

(35) $$\begin{array}{c} \sigma \left(\rho \right)=\sigma \;\left((1-\epsilon )\rho +\epsilon |0\rangle \langle 0|\right)=\left(1-\epsilon \right)\sigma \left(\rho \right)+\epsilon \sigma \left(|0\rangle \langle 0|\right), \end{array}$$

where linearity has been used. Since the input state $|\psi_{\mathrm{in}}\rangle$ is pure, the fidelity between the input and output states can be written as

(36) $$\begin{array}{cc} F(|\psi_{\mathrm{in}}\rangle ,\sigma (\rho ))\; & =\langle \psi_{\mathrm{in}}|\sigma (\rho )|\psi_{\mathrm{in}}\rangle \end{array}$$

(37) $$\begin{array}{cc} & =\left(1-\epsilon \right)F_{\mathrm{qubit}}+\epsilon F_{\mathrm{qutrit}}, \end{array}$$

where $F_{\mathrm{qubit}}$ denotes the fidelity associated with the encoded qubit subspace and $F_{\mathrm{qutrit}}$ corresponds to the contribution from the |0〉 component.

When the noise level is small (ϵ≤0.05), the state remains largely confined to the qubit subspace and the overall fidelity is dominated by $F_{\mathrm{qubit}}$, allowing the HQAM to outperform direct qutrit cloning. As the noise increases (0.05≤ϵ≤0.17), the encoded qubit component becomes increasingly distorted, leading to a gradual decline in cloning fidelity. For sufficiently large noise (ϵ≥0.17), the state becomes dominated by the |0〉 component, which the decoder reconstructs accurately. In this regime, the overall fidelity is therefore governed primarily by $F_{\mathrm{qutrit}}$, explaining the observed increase in fidelity.

**Figure 4:**
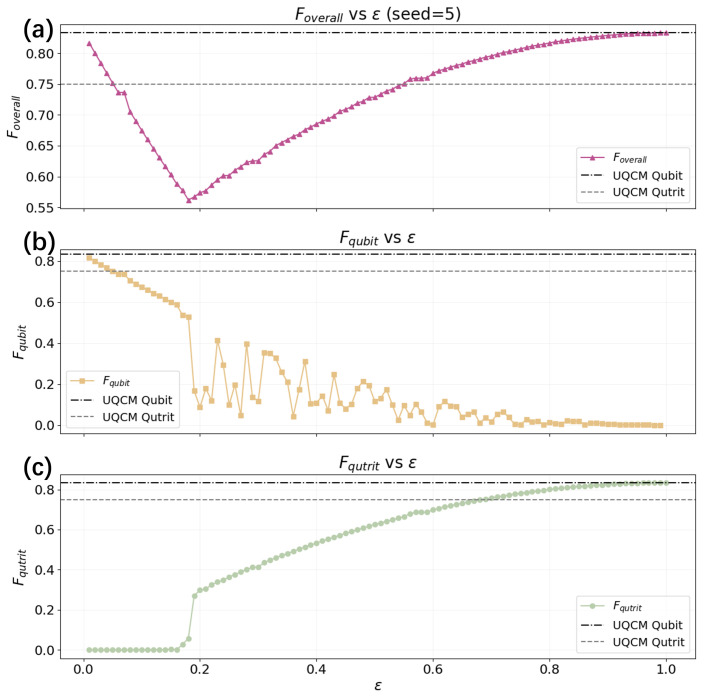
Performance of the HQAM with the leakage channel, where ϵ∈[0.01,1.00]. The two horizontal dashed lines are the average fidelities achieved by UQCM for qubit and qutrit, at $\frac{5}{6}$ and $\frac{3}{4}$, respectively. (**a**) The overall fidelity of the HQAM. (**b**) The fidelity of the effective qubit subspace. (**c**) The fidelity of the qutrit subspace.

Figure 4 further illustrates the contributions from the qubit and qutrit components. As ϵ increases, the fidelity associated with the qubit subspace $F_{\mathrm{qubit}}$ deteriorates due to the increasing leakage from the encoded subspace. In contrast, the fidelity associated with the |0〉 component, $F_{\mathrm{qutrit}}$, improves as the noise channel increasingly biases the state toward |0〉, which the decoder reconstructs reliably. Importantly, the overall fidelity remains bounded by the theoretical limits imposed by universal quantum cloning machines. For qubits, the optimal fidelity is F=5/6, while for qutrits, it is F=3/4. The fact that the observed fidelities never exceed these limits confirms that the HQAM remains fully consistent with the no-cloning theorem.

## 4. Discussions and Conclusions

In this work, we proposed a Hybrid Quantum Autocloning Machine that integrates quantum autoencoding with universal quantum cloning. By compressing qutrit states into an effective qubit subspace through a unitary encoder, $U_{e}$, the protocol enables cloning to be performed in a lower-dimensional space before reconstructing the state in the original Hilbert space. This compression-assisted strategy reduces the effective resource requirements while remaining fully consistent with the limits imposed by the no-cloning theorem. The encoder plays the role of a sample-based, tomography-free realisation of quantum principal component analysis on the input ensemble: it converges to the principal component projector of the empirical average state $\bar{\rho }$.

An illustrative photonic encoding model is provided in Appendix A. Numerical optimisation of the encoder confirms that the HQAM achieves high cloning fidelities when the input states have a strong overlap with the effective qubit subspace. In particular, for χ above the analytical crossover $\chi_{\mathrm{crit}}\geq 3/\sqrt{10}$, the achieved fidelity exceeds the benchmark fidelity of direct qutrit UQCM cloning and approaches the optimal qubit cloning limit as χ→1. On Haar-scrambled ensembles where the latent subspace is unknown to the encoder, the trained HQAM saturates the analytical maximum ${\bar{F}}_{\max}\left(\chi \right)=\left(5/6\right)\chi^{2}$ predicted by the principal component projector, while a fixed canonical isometry that lacks knowledge of the latent subspace cannot solve the problem and sits at the Haar-random scramble limit 5/9. We further analyse the robustness of the protocol under a leakage noise model, which shows that increasing noise degrades the fidelity associated with the encoded qubit component, while the contribution from the reconstructed |0〉 state becomes dominant at large ϵ. This behaviour explains the non-monotonic fidelity trends observed in the simulations and highlights the role of the encoded subspace in determining the overall cloning performance.

Overall, the HQAM demonstrates how compression techniques can be combined with approximate quantum cloning to improve cloning performance for structured high-dimensional quantum states whose latent subspace is not classically available. Future work could extend this framework to higher-dimensional systems, alternative cloning protocols such as phase-covariant cloning [21], and experimental implementations on photonic or near-term quantum computing platforms [6,22,23,24,25,26,27].

## Figures and Tables

**Table 1:** Held-out fidelity statistics of the trained HQAM encoder at β=8.0, η=0.01, aggregated over 50 runs per χ (10 Haar-random scrambles × 5 weight initialisations). The fourth column gives the analytical prediction $\left(5/6\right)\chi^{2}$ from Equation (19); the gap column reports the residual $\left(5/6\right)\chi^{2}-{\bar{F}}_{\mathrm{HQAM}}$, consistent with finite-sample noise across all χ.

χ	${\bar{F}}_{\mathrm{HQAM}}$ (Mean)	Std	$\left(5/6\right)\chi^{2}$ (Equation (19))	Gap
0.850	0.60232	$2\times 10^{-4}$	0.60208	$-2.4\times 10^{-4}$
0.875	0.63792	$9\times 10^{-5}$	0.63802	$1.0\times 10^{-4}$
0.900	0.67504	$1\times 10^{-4}$	0.67500	$-4\times 10^{-5}$
0.925	0.71279	$1\times 10^{-4}$	0.71302	$2.3\times 10^{-4}$
0.950	0.75214	$3\times 10^{-5}$	0.75208	$-6\times 10^{-5}$
0.975	0.79214	$2\times 10^{-5}$	0.79219	$5\times 10^{-5}$
0.990	0.81677	$1\times 10^{-5}$	0.81675	$-2\times 10^{-5}$

**Figure A1:**
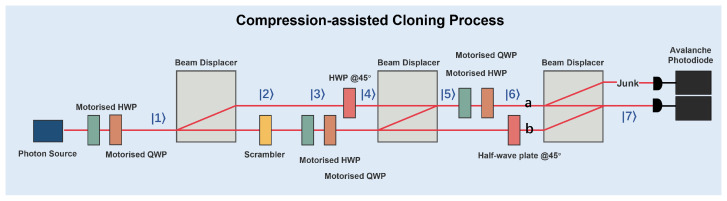
Schematic of the compression-assisted encoding process. The photon state at each intermediate step is labelled. Diagram modified from Ref. [17].

**Figure A2:**
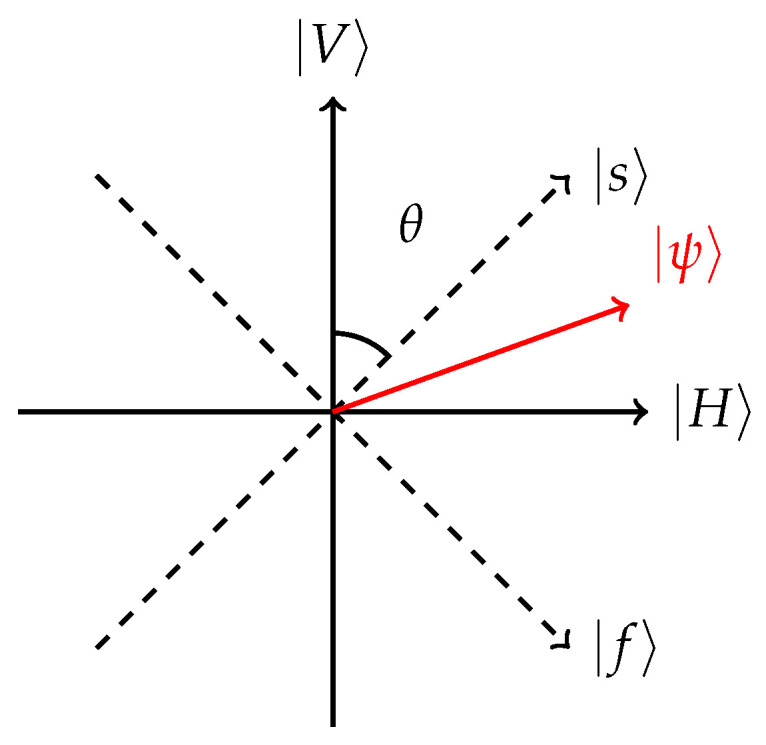
Visualisation of the basis transformation from the standard polarisation basis {|H〉,|V〉} to the principal basis {|f〉,|s〉}. The solid black horizontal and vertical axes denote the original {|H〉,|V〉} basis directions. The dashed black diagonal axes denote the rotated principal directions associated with the slow and fast axes, {|s〉,|f〉}. The angle θ indicates the rotation relating the two bases. The red vector represents the photon state |ψ〉, shown in the same plane so that its components can be resolved in the principal basis.

**Figure A5:**
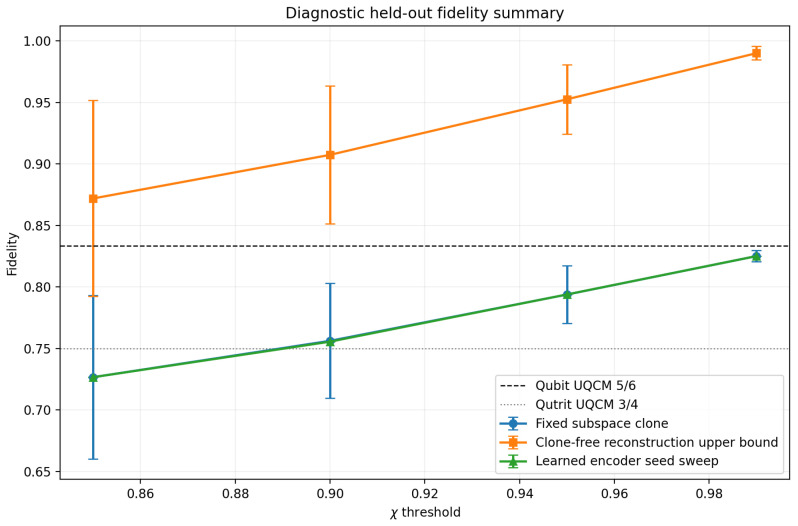
Diagnostic held-out fidelity summary for the structured pseudo-test ensembles. The figure compares the learned HQAM repeated-seed evaluation with internal fixed-subspace and clone-free reconstruction diagnostics, together with the universal qutrit and qubit cloning reference fidelities. This diagnostic figure is provided to contextualise the structured-prior setting and should not be interpreted as evidence of superiority over all fixed-subspace or state-dependent qutrit cloning strategies.

## Data Availability

The data underlying the results presented in this paper are available from the corresponding authors upon reasonable request.

## Appendix A. Illustrative Photonic Encoding Model

The main text focuses on the abstract HQAM model and its numerical implementation, while the photonic realisation is presented only as background material. Building on the information previously, we now analyse the compression-assisted cloning protocol in detail. The overall process of the analytical model for photonic implementation of the quantum autoencoder is illustrated in Figure A1. For convenience, we express the photon state in the fast (|f〉) and slow (|s〉) polarisation bases. The relation to the horizontal (|H〉) and vertical (|V〉) bases is given in Appendix B. The initial state produced by the single-photon source can therefore be written as

(A1) $$\begin{array}{c} |i\rangle =\cos\theta |f\rangle +\sin\theta |s\rangle , \end{array}$$

where θ is the polarisation parameter that determines the superposition between the fast and slow axes, and |f〉 and |s〉 denote the fast and slow polarisation axes, respectively. The encoding procedure consists of a sequence of optical transformations implemented by waveplates and beam displacers, whose detailed operations are summarised in Appendix B.

After passing through a half-wave plate (HWP) and a quarter-wave plate (QWP), the photon state becomes

$$|1\rangle =\cos\theta {|f\rangle }_{2}-i\sin\theta {|s\rangle }_{2},$$

where subscripts 1 and 2 denote the upper and lower paths. The beam displacer then separates the polarisation components into two spatial paths, resulting in the state,

$$|2\rangle =\cos\theta {|f\rangle }_{1}-i\sin\theta {|s\rangle }_{2},$$

A polarisation scrambler is subsequently applied to the lower path, producing

$$|3\rangle =\cos\theta {|f\rangle }_{1}-i\sin\theta \left(\cos\phi {|f\rangle }_{2}+\sin\phi {|s\rangle }_{2}\right),$$

where ϕ denotes the scrambler rotation angle. The encoding stage then recombines and rotates the polarisation components using additional waveplates. After the action of an HWP at $45^{\circ }$, the photon state becomes

$$|4\rangle =\cos\theta {|s\rangle }_{1}-i\sin\theta \left(\cos\phi {|f\rangle }_{2}-i\sin\phi {|s\rangle }_{2}\right).$$

Combining the polarisation components in the upper path yields

$$|5\rangle =\left(-i\sin\theta \cos\phi \right){|f\rangle }_{1}+\cos\theta {|s\rangle }_{1}-\sin\theta \sin\phi {|s\rangle }_{2}.$$

New spatial paths, *a* and *b*, are introduced, where path *a* corresponds to the recombined upper spatial mode and path *b* corresponds to the lower spatial mode. These paths represent physically distinct output ports after the polarisation-to-path encoding process. The photon state can be written as

$$|6\rangle =\left(-i\sin\theta \cos\phi \right){|f\rangle }_{a}-i\cos\theta {|s\rangle }_{a}-\sin\theta \sin\phi {|f\rangle }_{b}.$$

Finally, the mode corresponding to the discarded dimension is routed into the junk mode. The remaining encoded state is therefore

$$|7\rangle =-i\sin\theta \cos\phi {|f\rangle }_{c}-\sin\theta \sin\phi {|f\rangle }_{a}-i\cos\theta {|s\rangle }_{a}.$$

Here, *c* denotes the discarded spatial mode corresponding to the auxiliary dimension, which is routed into the junk mode.

The sequence of optical operations described above can be represented by a set of unitary transformations $U_{1},U_{2},\ldots ,U_{7}$ acting on the photon state. The states |i〉 to |4〉 describe a single-photon polarisation state together with path labels, while the four-component vector below is only a bookkeeping representation in the combined path–polarisation basis, rather than a generic four-level input state. Writing the input state in vector form,

(A2) $$\begin{array}{c} |\psi_{\mathrm{input}}\rangle =\left[\begin{array}{c} 0\\ 0\\ \cos\theta \\ -i\sin\theta \end{array}\right], \end{array}$$

Here, $|\psi_{\mathrm{input}}\rangle$ represents an effective state vector description of the photonic state in a chosen computational basis. Each optical element corresponds to a matrix operation on this state vector. The overall encoder transformation is therefore obtained by multiplying the individual unitaries,

(A3) $$\begin{array}{c} U_{e}=U_{7}U_{6}U_{5}U_{4}=\left[\begin{array}{cccc} 0 & 0 & 1 & 0\\ 0 & 1 & 0 & 0\\ 0 & 0 & 0 & -i\\ -i & 0 & 0 & 0 \end{array}\right]. \end{array}$$

To minimise the population of the junk mode (corresponding to path *c*), one may set θ=0, which suppresses the amplitude in that mode. Under this condition, the encoded state reduces to the qubit state

(A4) $$\begin{array}{c} |\psi_{\mathrm{out}}\rangle =-i|s\rangle . \end{array}$$

Applying the Bužek–Hillery universal quantum cloning machine (Equation (1)) to this encoded state yields

(A5) $$\begin{array}{c} |\psi_{\mathrm{out}}\rangle |R\rangle |M\rangle \to -i\sqrt{\frac{2}{3}}|s\rangle |s\rangle |f\rangle +i\sqrt{\frac{1}{6}}\left(|s\rangle |f\rangle +|f\rangle |s\rangle \right)|s\rangle . \end{array}$$

Expressing the resulting state in vector form gives

(A6) $$\begin{array}{c} |\psi_{\mathrm{cloned}}\rangle =i\sqrt{\frac{1}{6}}{\left[\begin{array}{cccccccc} 0 & 0 & 0 & 1 & 0 & 1 & -2 & 0 \end{array}\right]}^{\;⊤}. \end{array}$$

The density matrix of the output state is $\rho_{\mathrm{out}}=|\psi_{\mathrm{cloned}}\rangle \langle \psi_{\mathrm{cloned}}|$.

Tracing out the machine subsystem yields the reduced density matrices of the two clones

(A7) $$\begin{array}{c} \rho_{\mathrm{out}}^{\left(1\right)}=\rho_{\mathrm{out}}^{\left(2\right)}=\left[\begin{array}{cc} \frac{1}{6} & 0\\ 0 & \frac{5}{6} \end{array}\right]. \end{array}$$

The cloning fidelity is therefore $F=\frac{5}{6}$, which corresponds to the optimal fidelity for symmetric qubit cloning, as predicted by Equation (2).

## Appendix B. Transformation of Basis

The standard polarisation basis is {|H〉,|V〉}, while the alternative basis is defined by the fast and slow axes, denoted by {|f〉,|s〉}. Expressing the photon state in the principal basis simplifies the description of optical elements such as waveplates.

The transformation from the {|H〉,|V〉} basis to the {|f〉,|s〉} basis corresponds to a rotation (θ is the basis rotation angle)

(A8) $$\left[\begin{array}{c} \hat{f}\\ \hat{s} \end{array}\right]=\left[\begin{array}{cc} \cos\theta & -\sin\theta \\ \sin\theta & \cos\theta \end{array}\right]\left[\begin{array}{c} \hat{H}\\ \hat{V} \end{array}\right].$$

The inverse transformation is therefore

(A9) $$\left[\begin{array}{c} \hat{H}\\ \hat{V} \end{array}\right]=\left[\begin{array}{cc} \cos\theta & \sin\theta \\ -\sin\theta & \cos\theta \end{array}\right]\left[\begin{array}{c} \hat{f}\\ \hat{s} \end{array}\right].$$

Figure A2 illustrates this basis transformation and the corresponding photon state |ψ〉 in the rotated coordinate system. In our model, the fast-axis component can be represented as

(A10) $$\begin{array}{c} |f\rangle =\left(\begin{array}{c} \cos\theta \\ \sin\theta \end{array}\right), \end{array}$$

while the slow-axis component corresponds to the orthogonal state.

When the principal axes of a waveplate align with the chosen {|f〉,|s〉} basis, the corresponding transformations take particularly simple matrix forms. A half-wave plate (HWP) introduces a relative phase shift of π between the slow and fast components and is represented by

(A11) $$\begin{array}{c} {\mathrm{HWP}}_{\mathrm{principal}}=\left[\begin{array}{cc} 1 & 0\\ 0 & -1 \end{array}\right]. \end{array}$$

Similarly, a quarter-wave plate (QWP) introduces a phase shift of π/2 on the slow component, corresponding to

(A12) $$\begin{array}{c} {\mathrm{QWP}}_{\mathrm{principal}}=\left[\begin{array}{cc} 1 & 0\\ 0 & i \end{array}\right]. \end{array}$$

A half-wave plate oriented at $45^{\circ }$ exchanges the fast and slow components and can therefore be written as

(A13) $$\begin{array}{c} {\mathrm{HWP}}_{45}=\left[\begin{array}{cc} 0 & 1\\ 1 & 0 \end{array}\right]. \end{array}$$

Finally, the scrambler used in the encoding circuit mixes the polarisation components and can be modelled by

(A14) $$\begin{array}{c} \mathrm{Scrambler}=\left[\begin{array}{cc} 1 & 0\\ \cos\phi & \sin\phi \end{array}\right], \end{array}$$

where ϕ denotes the rotation angle of the scrambler.

## Appendix C. State Generation Procedure

In the numerical simulations, four ensembles of 2000 normalised qutrit states were generated at random. The parameter χ denotes the overlap between the qutrit state and the effective qubit subspace used by the encoder.

Four datasets were generated corresponding to different overlap thresholds, namely

χ≥0.99,χ≥0.95,χ≥0.90,χ≥0.85.

Random qutrit states satisfying a given overlap constraint were generated by sampling three complex coefficients

(A15) $$\begin{array}{c} c_{k}=x_{k}+iy_{k},\;x_{k},y_{k}∼N\left(0,1\right),\;k=0,1,2, \end{array}$$

followed by normalisation

(A16) $$\begin{array}{c} |\psi_{\mathrm{in}}\rangle =\frac{1}{\sqrt{|c_{0}{|}^{2}+|c_{1}{|}^{2}+{\left|c_{2}\right|}^{2}}}\left[\begin{array}{c} c_{0}\\ c_{1}\\ c_{2} \end{array}\right]. \end{array}$$

For simulations requiring a fixed overlap value χ=c, a modified procedure was used. First, the amplitude of the non-qubit component was chosen as

(A17) $$\begin{array}{c} A=\sqrt{1-\chi^{2}}. \end{array}$$

A random phase ϕ∼U(0,2π) was sampled, and the first coefficient was set to

(A18) $$\begin{array}{c} c_{0}=Ae^{i\phi }. \end{array}$$

Next, two complex numbers

(A19) $$\begin{array}{c} z_{k}=x_{k}+iy_{k},\;x_{k},y_{k}∼N\left(0,1\right),\;k=1,2 \end{array}$$

were generated and normalised to obtain

(A20) $$\begin{array}{c} {\tilde{z}}_{k}=\frac{z_{k}}{\sqrt{|z_{1}{|}^{2}+{\left|z_{2}\right|}^{2}}}. \end{array}$$

Finally, the coefficients in the qubit subspace were scaled according to the prescribed overlap,

(A21) $$\begin{array}{c} c_{1}=\chi {\tilde{z}}_{1},\;c_{2}=\chi {\tilde{z}}_{2}, \end{array}$$

which yields the state

(A22) $$\begin{array}{c} |\psi_{\mathrm{in}}\rangle =\left[\begin{array}{c} c_{0}\\ c_{1}\\ c_{2} \end{array}\right]. \end{array}$$

By construction, the state is normalised and satisfies

(A23) $$\begin{array}{c} |c_{0}{|}^{2}+|c_{1}{|}^{2}+{\left|c_{2}\right|}^{2}=1. \end{array}$$

## Appendix D. Optimised Encoder Unitaries

In this appendix, we report the encoder unitaries obtained from the optimisation procedure. The encoder $U_{e}$ was trained under four different overlap conditions between the qutrit Hilbert space and the effective qubit subspace. In all trials, the hyperparameters were fixed to β=8.0 and η=0.01. The optimisation was performed separately for datasets with overlap thresholds χ≥0.99, χ≥0.95, χ≥0.90, and χ≥0.85.

For the case χ≥0.99, the optimised encoder unitary is

$$U_{e}^{\left(0.99\right)}=\left[\begin{array}{ccc} -0.961721-0.2739745\;i & 0.004417+0.001720511\;i & -0.001957+0.001891414\;i\\ -0.004914-0.001463557\;i & -0.92631-0.2890803\;i & 0.238638-0.03752783\;i\\ -0.001791-0.0006179848\;i & 0.192974-0.1453321\;i & 0.43669-0.8665668\;i \end{array}\right].$$

For the case χ≥0.95, the optimised encoder unitary is

$$U_{e}^{\left(0.95\right)}=\left[\begin{array}{ccc} -0.309344+0.9509026\;i & -0.005232+0.00350046\;i & 0.007071-0.0007754599\;i\\ -0.002113+1.688474\times 10^{-4}\;i & -0.156878-0.5922081\;i & -0.024709-0.7899771\;i\\ 0.001987-0.009043409\;i & -0.665418+0.4264479\;i & 0.577064-0.2056022\;i \end{array}\right].$$

For the case χ≥0.90, the optimised encoder unitary is

$$U_{e}^{\left(0.90\right)}=\left[\begin{array}{ccc} -0.9954852-0.092547\;i & 0.01183747-0.001949\;i & 0.009575159+0.014441\;i\\ -0.01932928-0.005484\;i & -0.4346251-0.234607\;i & -0.4781819-0.725947\;i\\ -0.005968226+0.002199\;i & -0.0007486880+0.869434\;i & 0.2344573-0.434825\;i \end{array}\right].$$

For the case χ≥0.85, the optimised encoder unitary is

$$U_{e}^{\left(0.85\right)}=\left[\begin{array}{ccc} -0.9802706+0.1965275\;i & -0.002942577+0.02091174\;i & 0.0004458398-0.0004020995\;i\\ 0.01452004-0.008657617\;i & 0.1423112+0.7797590\;i & -0.3457613-0.5018831\;i\\ 0.003714416+0.01211348\;i & -0.6093131-0.003839584\;i & -0.7250697+0.3206806\;i \end{array}\right].$$

These matrices represent the encoder transformations learned by the HQAM for different input state distributions characterised by the overlap parameter χ.

## Appendix E. Depolarising and Leakage Channels

In this appendix, we briefly review the depolarising channel for a general *d*-dimensional quantum system and show that the leakage model used in the main text corresponds to a valid, completely positive, and trace-preserving (CPTP) quantum operation.

### Appendix E.1. Depolarising Channel in d Dimensions

For a *d*-dimensional quantum system with density matrix ρ, the depolarising channel is defined as

(A24) $$E\left(\rho \right)=\left(1-p\right)\rho +p\;\frac{I}{d},$$

where I denotes the identity operator and I/d is the maximally mixed state.

This channel can be expressed using the operator sum representation

(A25) $$E\left(\rho \right)=\sum_{k}K_{k}\rho K_{k}^{†},$$

where the Kraus operators satisfy the completeness relation

(A26) $$\sum_{k}K_{k}^{†}K_{k}=I.$$

A convenient choice of Kraus operators is

(A27) $$K_{0}=\sqrt{1-p}\;I,$$

and

(A28) $$K_{i}=\sqrt{\frac{p}{d^{2}-1}}\;E_{i},\;i=1,\ldots ,d^{2}-1,$$

where $\left\{E_{i}\right\}$ denotes an orthonormal basis of traceless operators satisfying

(A29) $$\mathrm{Tr}\left(E_{i}^{†}E_{j}\right)=\delta_{ij}.$$

With this choice, one obtains

(A30) $$E\left(\rho \right)=\left(1-p\right)\rho +\frac{p}{d^{2}-1}\sum_{i=1}^{d^{2}-1}E_{i}\rho E_{i}^{†}.$$

Using Schur’s lemma, we have

(A31) $$\sum_{i=1}^{d^{2}-1}E_{i}\rho E_{i}^{†}=I-\frac{1}{d}\rho .$$

Then we obtain

(A32) $$E\left(\rho \right)=\left(1-p\right)\rho +\frac{p}{d^{2}-1}\left(I-\frac{1}{d}\rho \right)=\left(1-p-\frac{p}{d(d^{2}-1)}\right)\rho +\frac{p}{d^{2}-1}I.$$

Since this expression does not recover the standard depolarising channel, we instead adopt the Kraus operators

(A33) $$K_{0}=\sqrt{1-p-\frac{p}{d^{2}}}\;I,\;K_{i}=\sqrt{\frac{p}{d}}\;E_{i},\;i=1,\ldots ,d^{2}-1,$$

### Appendix E.2. Special Case: Qubit Depolarising Channel

For a qubit system (d=2), the channel reduces to

(A34) $$E\left(\rho \right)=\left(1-p\right)\rho +p\;\frac{I}{2},$$

which is the form used in Equation (33) of the main text.

A common Kraus representation employs the Pauli operators $\left\{\sigma_{x},\sigma_{y},\sigma_{z}\right\}$,

(A35) $$K_{0}=\sqrt{1-p}\;I,\;K_{i}=\sqrt{\frac{p}{3}}\;\sigma_{i},\;i=x,y,z.$$

The completeness condition is verified as

(A36) $$\sum_{i}K_{i}^{†}K_{i}=I.$$

### Appendix E.3. Validity of the Leakage Channel

The leakage channel introduced in Equation (34) of the main text is

(A37)
E(ρ)=(1−ϵ)ρ+ϵ|0〉〈0|.

This channel describes population leakage from the encoded qubit subspace into a fixed qutrit state |0〉. To show that this mapping is a valid quantum operation, we construct the Kraus operators

(A38) $$R_{i}=|0\rangle \langle i|,\;i=0,\ldots ,d-1.$$

For any density matrix ρ,

(A39) $$\sum_{i=1}^{d}R_{i}\rho R_{i}^{†}=|0\rangle \langle 0|\;\mathrm{Tr}\left(\rho \right).$$

Since Tr(ρ)=1, this map replaces the input state by |0〉〈0|. The Kraus operators satisfy

(A40) $$\sum_{i}R_{i}^{†}R_{i}=\sum_{i}\left|i\rangle \langle i\right|=I.$$

Therefore, the leakage channel can be written as the convex combination

(A41) E(ρ)=(1−ϵ)ρ+ϵ|0〉〈0|,

which is a mixture of the identity channel and a replacement channel. Since both maps are CPTP, their convex combination is also CPTP. Hence, the leakage model used in the main text represents a valid quantum operation.

## References

[1] Bennett C.H., DiVincenzo D.P. (2000). Quantum information and computation. Nature.

[2] Nielsen M.A., Chuang I.L. (2010). Quantum Computation and Quantum Information.

[3] Gisin N., Thew R. (2007). Quantum communication. Nat. Photonics.

[4] Couteau C., Barz S., Durt T., Gerrits T., Huwer J., Prevedel R., Rarity J., Shields A., Weihs G. (2023). Applications of single photons to quantum communication and computing. Nat. Rev. Phys..

[5] Burger A., Kwek L.C., Poletti D. (2022). Digital quantum simulation of the spin-Boson model under Markovian open-system dynamics. Entropy.

[6] Zhan Y., Zhang H., Erbanni R., Burger A., Wan L., Jiang X., Chae S., Liu A.Q., Poletti D., Kwek L.C. (2026). Loop Quantum Photonic Chip for Coherent Multi-Time-Step Evolution. Laser Photonics Rev..

[7] Gross C., Bloch I. (2017). Quantum simulations with ultracold atoms in optical lattices. Science.

[8] Daley A.J., Bloch I., Kokail C., Flannigan S., Pearson N., Troyer M., Zoller P. (2022). Practical quantum advantage in quantum simulation. Nature.

[9] Lvovsky A.I., Sanders B.C., Tittel W. (2009). Optical quantum memory. Nat. Photonics.

[10] Julsgaard B., Sherson J., Cirac J.I., Fiurášek J., Polzik E.S. (2004). Experimental demonstration of quantum memory for light. Nature.

[11] Lau J.W.Z., Lim K.H., Shrotriya H., Kwek L.C. (2022). NISQ computing: Where are we and where do we go?. AAPPS Bull..

[12] Bharti K., Cervera-Lierta A., Kyaw T.H., Haug T., Alperin-Lea S., Anand A., Degroote M., Heimonen H., Kottmann J.S., Menke T. (2022). Noisy intermediate-scale quantum algorithms. Rev. Mod. Phys..

[13] Wootters W.K., Zurek W.H. (1982). A single quantum cannot be cloned. Nature.

[14] Scarani V., Iblisdir S., Gisin N., Acín A. (2005). Quantum cloning. Rev. Mod. Phys..

[15] Romero J., Olson J.P., Aspuru-Guzik A. (2017). Quantum autoencoders for efficient compression of quantum data. Quantum Sci. Technol..

[16] Bravo-Prieto C. (2021). Quantum autoencoders with enhanced data encoding. Mach. Learn. Sci. Technol..

[17] Pepper A., Tischler N., Pryde G.J. (2019). Experimental Realization of a Quantum Autoencoder: The Compression of Qutrits via Machine Learning. Phys. Rev. Lett..

[18] Lloyd S., Mohseni M., Rebentrost P. (2014). Quantum principal component analysis. Nat. Phys..

[19] Bužek V., Hillery M. (1996). Quantum copying: Beyond the no-cloning theorem. Phys. Rev. A.

[20] Jun B.Q. (2026). Quantum Autocloning Machine. https://github.com/01QJ10/ACM.

[21] D’Ariano G.M., Macchiavello C. (2003). Optimal phase-covariant cloning for qubits and qutrits. Phys. Rev. A.

[22] Hoch F., Rodari G., Caruccio E., Polacchi B., Carvacho G., Giordani T., Doosti M., Nicolau S., Pentangelo C., Piacentini S. (2025). Variational quantum cloning machine on an integrated photonic interferometer. Opt. Quantum.

[23] Zhan Y., Zhang H., Burger A., Cai H., Polett D., Kwek L., Liu A. (2024). Highly Efficient Photonic Multiplexing Chip Using Low-Loss Silicon Nitride Recirculating Loops. Conference on Lasers and Electro-Optics (CLEO).

[24] Wang H., Ralph T.C., Renema J.J., Lu C.Y., Pan J.W. (2025). Scalable photonic quantum technologies. Nat. Mater..

[25] Zhang R., Lou Y., Liu X., Wang J., Liu S., Jing J. (2025). Experimental demonstration of deterministic all-optical quantum cloning with phase-conjugate inputs. Photonics Res..

[26] Pelofske E., Bärtschi A., Eidenbenz S., Garcia B., Kiefer B. (2024). Probing quantum telecloning on superconducting quantum processors. IEEE Trans. Quantum Eng..

[27] Zhan Y., Zhang H., Wan L., Karim M., Cai H., Kwek L., Liu A. (2022). A hybrid quantum computer for quantum finance computation of Monte-Carlo distribution. Proceedings of the Novel Optical Materials and Applications.

